# The Role of Glial Mitochondria in α-Synuclein Toxicity

**DOI:** 10.3389/fcell.2020.548283

**Published:** 2020-11-11

**Authors:** Yu-Mi Jeon, Younghwi Kwon, Myungjin Jo, Shinrye Lee, Seyeon Kim, Hyung-Jun Kim

**Affiliations:** ^1^Dementia Research Group, Korea Brain Research Institute, Daegu, South Korea; ^2^Department of Brain and Cognitive Sciences, DGIST, Daegu, South Korea

**Keywords:** alpha-synuclein, mitochondrial dysfuncion, neurodegenerative diseases, non-cell autonomous, glia, neuron

## Abstract

The abnormal accumulation of alpha-synuclein (α-syn) aggregates in neurons and glial cells is widely known to be associated with many neurodegenerative diseases, including Parkinson’s disease (PD), Dementia with Lewy bodies (DLB), and Multiple system atrophy (MSA). Mitochondrial dysfunction in neurons and glia is known as a key feature of α-syn toxicity. Studies aimed at understanding α-syn-induced toxicity and its role in neurodegenerative diseases have primarily focused on neurons. However, a growing body of evidence demonstrates that glial cells such as microglia and astrocytes have been implicated in the initial pathogenesis and the progression of α-Synucleinopathy. Glial cells are important for supporting neuronal survival, synaptic functions, and local immunity. Furthermore, recent studies highlight the role of mitochondrial metabolism in the normal function of glial cells. In this work, we review the complex relationship between glial mitochondria and α-syn-mediated neurodegeneration, which may provide novel insights into the roles of glial cells in α-syn-associated neurodegenerative diseases.

## Introduction

Alpha synuclein (α-syn), first identified in 1988 by Maroteaux et al. (1988), is a small protein that consists of 140 amino acids and encodes the human *SNCA* gene. α-syn is expressed in the central nervous system (CNS), and is specifically localized in synapses and nuclei (Jakes et al., 1994). The function of α-syn is not clearly defined, but several studies have shown that α-syn regulates synaptic plasticity and vesicle trafficking (Kahle et al., 2000; Fortin et al., 2004; Lee et al., 2008) and interacts with synaptic vesicles to physiologically regulate vesicle recycling (Maroteaux et al., 1988; Huang et al., 2019). Originally, α-syn was thought to be a natively unfolded monomeric protein, but recent studies have indicated that α-syn forms an α-helix-rich tetramer (Bartels et al., 2011; Wang et al., 2011, 2014; Dettmer et al., 2013, 2015; Gurry et al., 2013; Burre et al., 2014). The biological function of α-syn is exerted through the N-terminal, non-amyloid-beta component (NAC) and C-terminal domains. The N-terminus, which contains the KTKEGV motif, maintains tetramerization of α-syn, and mutations in this motif can induce neurotoxicity (Dettmer et al., 2015). NAC, first identified in Alzheimer’s disease patients, is a highly hydrophobic domain and forms a β-sheet structure for α-syn aggregation (Ueda et al., 1993). The C-terminus of α-syn is a proline-rich region. α-syn can interact with other proteins through this domain (Kim et al., 2002). Unfolded or misfolded α-syn forms fibrillar aggregates and aggregated α-syn generates insoluble inclusions in the affected neurons and glial cells of α-Synucleinopathy brains.

Accumulated α-syn is regarded as a key feature of α-Synucleinopathy. In many neurodegenerative diseases, such as Parkinson’s disease (PD), dementia with Lewy bodies (DLB), and multiple system atrophy (MSA), show an α-Synucleinopathy phenotype. Intercellular delivery of α-syn in α-Synucleinopathy occurs through direct penetration, endocytosis, nanotube tunneling-mediated pore formation, and diffusion (Ubhi et al., 2011; Konno et al., 2012; Tosatto et al., 2012; Dieriks et al., 2017; Eguchi et al., 2017). Cell-to-cell delivery of α-syn contributes to neurodegeneration (Desplats et al., 2009; Bruck et al., 2016). Many studies have provided evidence that α-syn transmission occurs in a prion-like manner (Bernis et al., 2015; Steiner et al., 2018). However, this finding remains controversial because of the incomplete understanding of the factors that control the spread of pathogenic proteins and how they work.

Aggregated α-syn leads to many pathological features, such as mitochondrial dysfunction (Liu et al., 2009; Cali et al., 2012; Vicario et al., 2018), dysregulation of calcium homeostasis (Cali et al., 2012; Melachroinou et al., 2013), neuroinflammation, endoplasmic reticulum (ER) stress, protein quality control impairment (Melo et al., 2018; Rocha et al., 2018), Golgi fragmentation (Gosavi et al., 2002), and lysosomal dysfunction (Meredith et al., 2002). In MSA, unlike PD and DLB, aggregated α-syn mainly appears in oligodendrocyte, also called glial cytoplasmic inclusions (GCIs; Ahmed et al., 2013; Bruck et al., 2016; Mori et al., 2020). Gila cells are non-neuronal cells in brain and play a critical role in maintaining neuronal system. The majority of brain cells are glial cells, and they modulate neurogenesis and synaptogenesis (Argente-Arizon et al., 2017). Moreover, glial cells affect brain-blood barrier (BBB) development and function through interactions with neurons and endothelial cells to defend the brain from pathogens (Banerjee and Bhat, 2007; Broux et al., 2015). Glia cells are composed of astrocytes, microglia, and oligodendrocytes in the CNS (Bruck et al., 2016). A major function of astrocytes and microglia involve the immune response. Under pathological conditions, microglia and astrocytes are activated. Activated microglia and astrocytes can release pro-inflammatory cytokines such as interleukin-1 (IL-1), tumor necrosis factor alpha (TNF-α), and interleukin-6 (IL-6; Liu et al., 2011; Tjalkens et al., 2017). These cytokines lead to the production of reactive oxidative stress (ROS) and dysfunction of the BBB. Finally these factors induce neuronal cell death (Yang et al., 2007; Pan et al., 2011) and cause neurodegenerative disease. In general, protein aggregation in affected neurons is also detected in glial cells (Li and Haney, 2020). In particular, α-syn inclusions in glial cells causes a reduction in trophic support, which in turn leads to neuronal loss (Bruck et al., 2016).

Protein aggregation generally occurs in neurodegenerative diseases. In glial cell, aggregated proteins have also been observed (Li and Haney, 2020). Currently, many studies emphasize the role of mitochondria in glia (Yang et al., 2017; McAvoy and Kawamata, 2019; Yan et al., 2020). The mitochondria of glial cells regulate calcium homeostasis, ATP production and the inflammatory response (Yang et al., 2017). Mitochondrial impairment in glia can affect neuronal survival, so glial mitochondrial dysfunction has recently emerged as a major etiology of neurodegeneration (Yang et al., 2017; McAvoy and Kawamata, 2019; Yan et al., 2020).

In this review, we provide new insights into the roles of glial cells in α-syn-related neurodegenerative diseases and examine the importance of glial mitochondria in the disease progression of α-Synucleinopathy.

## Neurodegenerative Disorders Associated With α-Synucleinopathies

### PD

Parkinson’s disease is the second most common neurodegenerative disease and is defined as a α-Synucleinopathy. The main pathological features of PD are the selective loss of dopaminergic neurons in the substantia nigra pars compacta (SNpc; Damier et al., 1999; Kalia and Lang, 2015) and protein aggregates (called Lewy bodies and Lewy neurites) consisting mainly of α-syn proteins present in neurons (Spillantini et al., 1997; Gomez-Benito et al., 2020). Moreover, PD has clinical features such as cognitive impairment and motor dysfunction including stiffness, postural instability, and akinesia. PD is mostly known as a sporadic disease, but several gene mutations, such as α-syn, LRRK2, PINK1, DJ-1, and parkin, cause disease (Davie, 2008). Moreover, mutations in α-syn (duplications, triplications, or point mutations) facilitate aggregate formation in neurons and affect the exacerbation and progression of PD. Many pathological features of PD, such as mitochondrial dysfunction (Park et al., 2018; Wang X.L. et al., 2020), neuroinflammation (Wang et al., 2015; Pajares et al., 2020), ER stress (Colla, 2019), and impaired protein quality control (Cook and Petrucelli, 2009; Sala et al., 2016), can lead to neuronal toxicity. α-Synucleinopathy features the accumulation of aggregated α-syn in neuronal and glial cells (McCann et al., 2014). Previous studies showed that healthy neurons transplanted into the striatum of PD patient exhibited α-syn pathology (Kordower et al., 2008; Li et al., 2008). These results indicated that α-syn can propagate into other cells. Moreover, cell-to-cell transmission of aggregated α-syn accelerates Synucleinopathy. Mitochondrial dysfunction is a key feature in PD progression, which is caused by disrupted mitochondrial respiration, decreased mitochondrial membrane potential, and impaired mitophagy (Lin et al., 2019). Moreover, α-syn also influences mitochondrial function (Devi et al., 2008; Pozo Devoto and Falzone, 2017). α-Synucleinopathy induces mitochondrial dysfunction, especially in the context of calcium homeostasis (Vicario et al., 2018; Lin et al., 2019). Mitochondria regulate calcium levels through interactions with ER. However, abnormal α-syn accumulates in both the mitochondria and ER and stresses the ER to release too much calcium into mitochondria. Excessive calcium in mitochondria induces ROS, which can lead to cell death (Melo et al., 2018; Lin et al., 2019). In addition, α-syn is associated with another mitochondrial dynamics, such as the regulation of morphology (fission and fusion), axonal transport and mitophagy (Pozo Devoto and Falzone, 2017).

### DLB (Dementia With Lewy Bodies)

Lewy body dementia (LBD) consists of DLB and Parkinson’s disease dementia (PDD), and it shares pathological characteristics with PD, such as the accumulation of α-syn, leading to neuronal loss (Kim et al., 2014; Gomperts, 2016; Surendranathan et al., 2020). These patients commonly show parkinsonism motor symptoms, neuropsychiatric symptoms, cognitive defects, sleep disorders, and visual hallucinations (McKeith et al., 2017; Velayudhan et al., 2017). Moreover, depressive symptom is also common in DLB patients. Several studies indicated that depression is associated with AD and DLB which cause cognitive and memory dysfunction (Fritze et al., 2011; Rapp et al., 2011). However, the frequency and severity of depression are more severe DLB than AD (Fritze et al., 2011; Chiu et al., 2017). Although LBD and PD have some common features, such as α-syn aggregates and clinical symptoms, they are distinguished by the relative onset and prognosis. DLB is diagnosed as dementia within 1 year after parkinsonian onset. On the other hand, PDD is diagnosed with dementia 1 year after parkinsonian onset. PDD patients show severe movement dysfunctions, whereas DLB patients show severe cognitive impairments (Hansen et al., 2019). DLB is caused by the abnormal accumulation of α-syn in neurons, called Lewy bodies (LBs) and Lewy neurites (LNs; Cummings, 2004; Walker et al., 2015). Most DLB cases occur sporadically, but several papers have suggested that DLB has a genetic cause, such as APOE, SNCA, and LRRK2 (Walker et al., 2015; Orme et al., 2018). Excessive accumulation of α-syn in DLB is associated with the loss and dysfunction of dopaminergic neurons and cholinergic neurons (Tiraboschi et al., 2000; Gomperts, 2016). Cholinergic neurons participate in memory function and age-related dementia including Alzheimer’s disease (Haam and Yakel, 2017). In addition to LB and LN pathology, most DLB patients also have amyloid plaque and neurofibrillary pathology in their brains (Perry et al., 1993; Bohnen and Albin, 2011). Unfortunately, the clinical diagnosis of DLB remains difficult. Because the symptoms of DLB are similar to those of other diseases, such as AD, PD, and PDD (Tousi, 2017), DLB symptoms can overlap with those of AD and PDD at the same time (McKeith et al., 2017). Therefore, DLB is usually diagnosed after death.

### MSA (Multiple System Atrophy)

Multiple system atrophy is an uncommon neurodegenerative disease that is pathologically characterized by a combination of parkinsonism, cerebellar ataxia, and autonomic dysfunction (Lopez-Cuina et al., 2018; Monzio Compagnoni and Di Fonzo, 2019). MSA is divided into two subtypes, MSA with parkinsonism features (MSA-P), which exhibits bradykinesia, rigidity, postural instability, and tremor, or MSA with cerebellar features (MSA-C), which exhibits gait ataxia, ataxic dysarthria, limb ataxia, and sustained gaze-evoked nystagmus (Gilman et al., 1999). MSA is caused by the aggregation of α-syn in oligodendrocytes, which are called GCIs and are regarded as a key feature of this disease (Asi et al., 2014; Djelloul et al., 2015; Monzio Compagnoni and Di Fonzo, 2019). Moreover, one case report suggested that α-syn co-localize with phosphorylated tau in GCIs in MSA patients (Piao et al., 2001). Normally, oligodendrocytes exhibit very low-level expression of α-syn (Miller et al., 2005). The mechanisms of α-syn accumulation in oligodendrocytes are not fully understood. Recently, some researchers suggested that the interaction between neurons and oligodendrocytes affects GCIs. The overexpression of α-syn in neurons induces extracellular secretion of α-syn. Then, oligodendrocytes absorb α-syn into the cell in clathrin-dependent manners. In addition, intracellular aggregated α-syn in neurons also increases α-syn release from neurons and this process is mediated by vesicle trafficking (Kisos et al., 2012; Konno et al., 2012; Monzio Compagnoni and Di Fonzo, 2019). Originally, prion-like α-syn propagation was examined in PD (Chu and Kordower, 2015). Monomeric α-syn aggregates into β-sheet structure, which are important features of the self-propagation of fibrillar α-syn aggregates (Chu and Kordower, 2015; Woerman et al., 2018). There are several pathological features of MSA, such as inflammation (Ishizawa et al., 2004; Jellinger, 2014) and impairments in protein degradation (Schwarz et al., 2012; Tanji et al., 2013). Moreover, mitochondrial impairment is implicated in MSA pathogenesis. A previous study showed that the coenzyme Q2 (COQ2) mutation induces MSA. The COQ2 mutation inhibits the synthesis of COQ10, which located in the mitochondrial inner membrane. COQ10 plays a role in the mitochondrial respiratory chain. Thus, loss-of-function of COQ10 increases oxidative stress and induces apoptotic cell death (Quinzii and Hirano, 2010; Multiple-System Atrophy Research Collaboration, 2013). It is already known that oxidative stress is a major contributor to the loss of oligodendrocytes (Bradl and Lassmann, 2010).

### PAF (Pure Autonomic Failure)

Unlike PD, DLB, and MSA, Pure autonomic failure (PAF) is a rare sporadic neurodegenerative disease induced by dysfunction of autonomic nervous system (Thaisetthawatkul, 2016). It is slowly progressive disease and caused by abnormal accumulation of α-syn (Thaisetthawatkul, 2016; Coon et al., 2019; Coon, 2020). PAF patients showed orthostatic hypotension, syncope, falls, sexual and bladder dysfunction, and constipation with no motor dysfunction (Stubendorff et al., 2012; Allan, 2019). The pathological characteristic of PAF is the degeneration of peripheral autonomic neurons which have α-syn aggregates (Singer et al., 2017). Furthermore, accumulation of cytoplasmic α-syn inclusion in brainstem nuclei contributes to PAF (Garland et al., 2013; Coon et al., 2019). Donadio et al. (2013) suggested that α-syn aggregation founded in sympathetic nerve fiber using skin biopsy, it might be a biomarker for PAF diagnosis. However, α-syn induced autonomic dysfunction is not clearly understood and there is no cure for PAF (Thaisetthawatkul, 2016). Recent studies suggested that abnormal α-syn in autonomic neurons of PAF patients is able to propagate into central nerve. Wang X.L. et al. (2020, Wang X. J. et al. 2020) reported that injection of α-syn preformed fibrils (PFF) into stellate and celiac ganglia in mutant form α-syn-expressing mice produces propagation of α-syn throughout autonomic pathway into CNS. They also showed that the PFF injected mice have PAF-related pathology. Moreover, about 70% of PAF patients showed rapid eye movement sleep behavior disorder (RBD) strongly associated with the function of the CNS (“Consensus statement on the definition of orthostatic hypotension, PAF, and MSA. The Consensus Committee of the American Autonomic Society and the American Academy of Neurology” No authors listed, 1996; Coon et al., 2019). Besides, more than 10% of patients initially diagnosed with PAF developed CNS diseases such as PD, DLB, and MSA (Muppidi and Miglis, 2017; Singer et al., 2017). Converting ratio of PAF to CNS diseases is twice as higher MSA than PD or DLB, and it might be associated with low circulating level of norepinephrine in the plasma of MSA and PAF patients (Goldstein et al., 1989).

**FIGURE 1:**
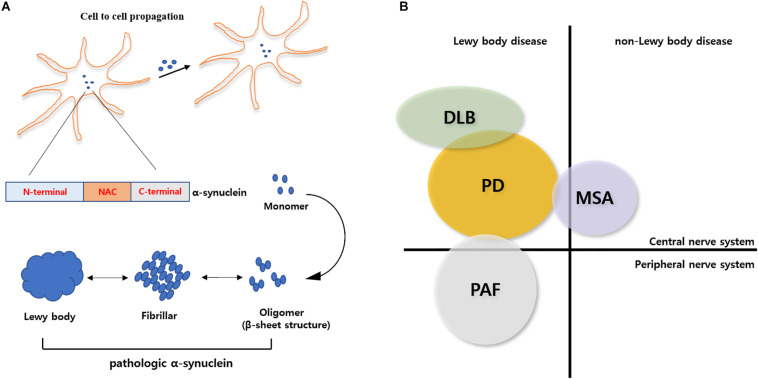
Schematic overview of α-synuclein physiology and pathology. **(A)** Pathological conformation change of α-syn. α-syn composed of N-terminal, NAC, and C-terminal. Originally, α-syn exist as native or misfolded monomeric state. Monomeric α-syn can aggregate into cytotoxic oligomer or fibrillar α-syn. Aggregated α-syn forms Lewy body and it can propagate into other cells. **(B)** α-Synucleinopathies in neurodegenerative diseases. Aggregated α-syn is associated many neurodegenerative diseases such as Parkinson’s disease, Dementia with Lewy Body, Pure Autonomic Failure and Multiple System Atrophy. PD, DLB and PAF belong to Lewy body disease.

The accumulation of α-syn aggregates might be the major cause of neurodegeneration in α-Synucleinopathy (Figure 1). Thus, there are several α-syn-mediated therapeutic approaches, such as prevention of α-syn aggregates, increasing of α-syn degradation and α-syn immunotherapy, in all α-syn-mediated diseases (Brundin et al., 2017).

## α-Synuclein-Induced Pathological Changes in Glial Cells

### Oligodendroglia (Oligodendrocytes)

Oligodendrocytes, together with astrocyte and microglia, are glial cells in CNS (Jakel and Dimou, 2017). Oligodendrocytes are myelin-forming cells in the CNS, accounting for approximately 5–8% of all cells in the adult brain (Levine et al., 2001). Myelin is essential for the transmission of electrical signals along the axon and additionally plays a role in supporting neuronal activity by regulating axon and neuro homeostasis through the supply of neurotrophic factors (Dawson, 2003; Wilkins et al., 2003; Bean, 2007; Peferoen et al., 2014; Kuhn et al., 2019). Demyelination can be caused by damage to oligodendrocytes or the myelin sheaths maintained by these cells (Arenella and Herndon, 1984); moreover, demyelination induces axonal degeneration and oligodendrocyte death, ultimately promoting neuronal death (Bradl and Lassmann, 2010; Kuhn et al., 2019). MS is a demyelination disease caused by inflammation in the CNS. In a previous study, overexpression of wild-type human α-syn in oligodendrocytes induced mitochondrial dysfunction in transgenic mice, leading to oxidative stress *in vivo* and neuronal cell death (Stefanova et al., 2005). The pathological features of oligodendrocytes are associated with ROS and the inflammatory response (Thorburne and Juurlink, 1996; Jurewicz et al., 2005). Normally, oligodendrocytes cooperate with astrocytes to regulate the immune response. A recent study suggested that nuclear factor κB (NF-κB) is a main inflammatory modulator in MS, and plays a role in myelin formation (Nickols et al., 2003; Stone et al., 2017). NF-κB predominantly localizes in the cytoplasm, and activated NF-κB translocate to the nucleus and activates the transcript of inflammatory genes including cytokines, chemokines, and adhesion genes (Lawrence, 2009). Moreover, another study showed that NF-κB activation in oligodendrocytes could drive experimental autoimmune encephalomyelitis (EAE) and the expression of interferon-γ (IFN- γ) to exacerbate the inflammatory response in an *in vivo* model (Lin et al., 2012, 2013; Stone et al., 2017). Additionally, oligodendrocytes contact surrounding nerve cells to provide neurotrophic factors such as glial cell line-derived neurotrophic factor (GDNF), brain-derived neurotrophic factor (BDNF), and insulin-like growth factor 1 (IGF-1) for protection (Bradl and Lassmann, 2010). Oligodendrocytes do not express α-syn under normal conditions (Kahle et al., 2002), but aggregated α-syn was found in oligodendrocytes in midbrains of PD and DLB patients (Wakabayashi et al., 2000). Furthermore, phosphorylated α-syn was observed in GCIs (Fujiwara et al., 2002). Abnormal accumulation of α-syn occurs with the demyelination and brain atrophy associated with neurodegeneration (Yoon et al., 2020). We already mentioned that MSA is caused by aggregated α-syn in GCIs. A recent paper showed that α-syn aggregation was observed in the oligodendrocytes of human MSA-P patients (May et al., 2014).

Several papers have shown that the aggregation of α-syn is accelerated by heparin and heparan sulfate (HS; Cohlberg et al., 2002; Ihse et al., 2017; Maiza et al., 2018). HS is a linear polysaccharide expressed on the cell surface or as an extracellular matrix protein (Iozzo, 1998; Medeiros, 2000). Heparan sulfate proteoglycans (HSPGs) are internalized by α-syn in the MO3.13 human oligodendrocyte cell line (Ihse et al., 2017). Furthermore, the presence of HSPGs initiates a conformational change in α-syn forms a native state into an oligomeric state, including β-sheet formation, through protein unfolding (Motamedi-Shad et al., 2009; Maiza et al., 2018). Aggregate α-syn uptake relies on HS by binding to the plasma membrane (Holmes et al., 2013; Ihse et al., 2017). Additionally, several hypotheses of α-syn aggregation exist cell-to-cell propagation of aggregated α-syn from neurons to oligodendrocytes and/or the secretion of α-syn by oligodendrocyte.

### Astroglia (Astrocytes)

Astrocytes are the most common type of glial cell in the CNS and account for at least one third of the brain mass. Astrocytes support the physiological function of the neuronal system by regulating blood flow, maintaining the blood-brain barrier and regulating synaptic plasticity (Sofroniew and Vinters, 2010; Chavarria et al., 2018). Moreover, astrocytes are the main cells responsible for glucose metabolism in the brain and release gliotransmitters, including γ-aminobutyric acid (GABA) and glutamate (Lee et al., 2010a). In addition, astrocytes also play major roles in maintaining neuronal homeostasis through the absorption of glutamate and potassium ions (Thorburne and Juurlink, 1996; Simard and Nedergaard, 2004).

A recent study found that astrocytes isolated from neuroinflammatory and ischemic brains showed two reactive phenotypes, A1 and A2 (Liddelow and Barres, 2017). A1 astrocytes are activated by pro-inflammatory microglia, impair synaptic formation, and kill both neurons and oligodendrocytes, while A2 astrocytes appear to produce substances that promote growth and neuronal survival. Although the number of A1 astrocytes are increased in the affected brain regions of many neurodegenerative diseases, including AD, HD, PD, ALS, and MS (Liddelow and Barres, 2017), detailed mechanisms of A1 and A2 induction have not been clearly identified.

In contrast to neurons, astrocytes express α-syn at very low levels. However, cytoplasmic immunoreactivity of α-syn in astrocytes has been clearly observed in the normal human brain (Mori et al., 2002). Some studies suggested that the number of reactive astrocytes did not increase in the SN and putamen regions in PD patients (Mirza et al., 1999; Song et al., 2009). However, other postmortem studies of PD patient brains reported massive increase in reactive astrocytes and the loss of dopaminergic neurons in the SN (Hirsch et al., 2005), while others reported that reactive astrogliosis was mild (Vila et al., 2001). In most postmortem studies using the brains of PD patients (Mythri et al., 2011; Lastres-Becker et al., 2012), increased expression of glial fibrillary acidic protein (GFAP) was used as a marker for reactive astrocytes (Eng et al., 1971; Dahl and Bignami, 1976). However, since a significant level of GFAP is already present in non-reactive astrocytes, it seems difficult to accurately measure the number of reactive astrocytes with GFAP immunostaining (Sofroniew and Vinters, 2010; Rusnakova et al., 2013).

Astrocytes also have phagocytic activity to degrade cellular debris and synaptic elements (Morizawa et al., 2017). Accumulating evidence suggests that the loss of proper phagocytic functions in astrocytes may contribute to neurodegenerative processes (Jones et al., 2013; Tong et al., 2014; Chung et al., 2016). Unfortunately, whether astrocytic receptors are involved in the recognition of α-syn aggregates and/or their delivery to lysosomes is still unknown.

Astrocytes respond to pathological stimuli by secreting inflammatory cytokines and increasing the level of GFAP. However, in the context of PD, it is still not clear whether astrocyte activation plays a positive or negative role in disease progression (Tremblay et al., 2019).

Both microglia and astrocytes can take up monomeric and fibrillar forms of α-syn (Stefanova et al., 2011; Fellner et al., 2013). In microglia, TLR4 has been implicated in α-syn clearance, the secretion of pro-inflammatory cytokines, and the production of ROS (Fellner et al., 2013). However, astrocytes do not require TLR4 for α-syn uptake (Fellner et al., 2013). Interestingly, α-syn oligomers are rapidly internalized by astrocytes, but internalized α-syn is not completely degraded by lysosomes. Thus, α-syn oligomers accumulate intracellularly and this accumulated α-syn induces mitochondrial defects (Lindstrom et al., 2017). Another study has also reported that the accumulation of α-syn in human astrocytes affects their phagocytic-lysosomal mechanism and that aggregated α-syn is transported through newly formed tunneling nanotubes (Rostami et al., 2017). Evidence suggests that astrocytic α-syn accumulation is implicated in neurotoxic alterations in astrocytes, but the underlying mechanism is still not clear.

### Microglia

Although microglia account for the smallest proportion (10% or less) of healthy mammalian brain glia (von Bartheld et al., 2016), they play an important role in the immune response in the CNS. Microglia also regulate neuronal activity and viability in the adult brain through direct contact with neurons (Li et al., 2012; Kohman et al., 2013) and the secretion of soluble factors (Ramirez et al., 2005; Tichauer et al., 2007).

The morphology and transformative ability of microglia are similar to those of peripheral macrophages/monocytes (Kreutzberg, 1996). The phagocytic activity of microglia is important for the developmental process, as well as injury repair in the adult brain (Neumann et al., 2009).

During brain development, a significant proportion of cells must be eliminated by programmed cell death, while the remaining cells differentiate and migrate. Microglia engulf dying cells, and this phagocytic activity contributes to the proper patterning of the developing brain (Marıìn-Teva et al., 2004; Frost and Schafer, 2016). In addition, microglia remove excess synapses in the developing brain and appear to play an important role in the wiring of the CNS (Jakel and Dimou, 2017). Under pathological conditions such as neurodegenerative diseases, infection and physical injury, microglia are activated. Activated microglia are polarized into two different states, M1 (pro-inflammatory), and M2 (anti-inflammatory), according to the signals in the surrounding environment and function in immune regulation (Moehle and West, 2015). M1 microglia release several pro-inflammatory cytokines, including TNF-α, IL-6, IL-12, and IL-1β, followed by CCL2 and CXCL10. In contrast to M1 cells, M2 microglia are associated with anti-inflammatory functions and promote wound healing and tissue repair. M2 microglia secrete major anti-inflammatory cytokines, such as IL-4, IL-13, IL-10, and TGF-β (Du et al., 2018). In PD, affected DA neurons in the brains of PD patients are surrounded by M1 microglia (Tang and Le, 2016). Moreover, microglia-mediated neuroinflammation shows an inverse relationship to the survival of dopaminergic neurons in PD patients (Tang and Le, 2016). Increased microglial activation has been predominantly found in the substantia nigra (SN) and other brain regions associated with pathological α-syn accumulation (Doorn et al., 2014). In addition, overexpression of the mutant α-syn in microglia induces polarization to the M1 phenotype, which is characterized by increased levels of pro-inflammatory cytokines (such as TNF-α and NO; Rojanathammanee et al., 2011). The role of M2 microglia in PD is not well understood, but the shift from the M1 pro-inflammatory state to the anti-inflammatory M2 state might be more beneficial in neuroprotection than simply blocking microglial activation (Subramaniam and Federoff, 2017). Thus, a therapeutic approach promoting M1 to M2 polarization could be promising in PD.

Toll-like receptors (TLRs) are known to be involved in the innate immune response, cell survival and death. TLRs are expressed in neurons (Tang et al., 2007), astrocytes (Bowman et al., 2003), microglia (Olson and Miller, 2004), and oligodendrocytes (Lehnardt et al., 2002) in the CNS. Many studies have reported an association between TLRs, especially TLR2, 4 and 9, and neurodegenerative diseases such as PD, AD and ALS. Several studies have shown that increased expression of various receptors, including TLR2 (Kim et al., 2013), TLR4 (Fellner et al., 2013), cluster of differentiation (CD) 11b (Hou et al., 2018), and CD36, (Su et al., 2008) by α-syn aggregation can lead to receptor-mediated activation of the inflammatory signaling cascade. TLR2 is one of the most well-studied TLRs in the context of neurodegenerative diseases. In mice, overexpression of α-syn increased TLR2 expression and microglial activation (Drouin-Ouellet et al., 2014).

The α-syn released by neurons acts as an endogenous ligand for TLR2 on microglia. TLR2-α-syn binding activates the inflammatory responses of microglia, which eventually produce toxic molecules that cause neurodegeneration (Kim et al., 2013, 2016). Previous studies have shown that TLR4 also plays an important role in α-Synucleinopathies.

Stimulation of TLR4 by monophosphoryl lipid A, a selective TLR4 agonist, in an α-syn-overexpressing mouse model, increased α-syn clearance by microglia and suppressed disease-like phenotype, such as motor deficiency (Venezia et al., 2017). In BV2 microglia-like cells, the TLR4-dependent immune response is modulated by Nurr1 and NF-κB signaling, and this TLR4-mediated neuroinflammation pathway is mediated by α-synuclein (Shao et al., 2019). In addition, Choi et al. (2020) showed that TLR4-NF-κB-p62-mediated selective autophagy activation in microglia was implicated in the clearance of neuron-secreted α-syn and this clearance mechanism was important for neuroprotective functions of microglia.

## Glial Mitochondrial Dysfunction in α-Synucleinopathies

### Cell Death

Severe mitochondrial damage can induce cell death via necrosis or apoptosis (Cillero-Pastor et al., 2011). Several studies have shown that the interaction between α-syn and cytochrome C oxidase (COX, mitochondrial complex IV) induces both α-syn aggregation and mitochondrial dysfunction, causing neurodegeneration (Irrinki et al., 2011; Al-Mansoori et al., 2013; Ciccone et al., 2013).

α-syn acts as a regulator of mitochondrial homeostasis, and mitochondrial deficiency and impairment are a key pathological features of PD (Gao et al., 2017; Pozo Devoto and Falzone, 2017). Complex 1 deficiency or dysfunction associated with mitochondrial dysfunction was found in the SN region of PD patients (Schapira et al., 1989; Parker et al., 2008). Furthermore, many α-Synucleinopathy models have shown that the level of α-syn expression is associated with cell death and mitochondrial deficiency (Hsu et al., 2000; Bellucci et al., 2008; Trancikova et al., 2012). Numerous studies have revealed that the interactions between mitochondria and α-syn play important roles in both physiological and pathological conditions, but most of these studies focused on their role in neurons (Faustini et al., 2017). However, some recent studies have suggested that α-syn-induced mitochondrial defects in glia are also implicated in various pathogenic features, such as neurodegeneration and neuroinflammation.

Accumulating evidence suggests that astrocytes might be a major contributor to the progression of α-Synucleinopathies. For example, α-syn overexpression in astrocytes induces astrocyte activation, and these reactive astrocytes cause neuronal death (Chavarria et al., 2018) and susceptibility to oxidative stress (Stefanova et al., 2001). Chavarria et al. (2018) investigated the effects of the different species of α-syn (monomer, oligomer, and fibrillar) protein on primary rat cortical astrocyte. All of α-syn species can activate astrocytes, and astrocytes treated with the different α-syn species induced cell death of hippocampal neuron in neuron-astrocyte co-culture. In particular, only oligomeric α-syn induce mitochondrial dysfunction and increased hydrogen peroxided generation in astrocytes, whereas fibrillar α-syn treated astrocytes enhanced the secretion of pro-inflammatory cytokines. Stefanova et al. (2001) showed that overexpression of wild-type α-syn or C-terminal truncated α-syn in U373 astrocytoma cells induced apoptotic cell death and increased susceptibility to oxidative stress, and that it was partially present in the cytoplasmic inclusion. In addition, *in vitro* and *in vivo* model studies have demonstrated that α-syn can spread from neurons to glial cells either through secretion to the extracellular space or direct cell-to-cell transfer (Angot et al., 2012; Lee et al., 2012; Reyes et al., 2015). α-syn inclusions are mainly found in neurons, but also frequently detected in glial cells. Lindstrom et al. (2017) tried to elucidate the clearance mechanism of toxic α-syn in glial cells. This study examined the uptake, degradation, and toxic effects of oligomeric α-syn in the co-culture system of primary mouse neurons, astrocytes, and oligodendrocytes. Astrocytes rapidly and extensively absorb α-syn from the extracellular space and sequester it. In the early stage of α-Synucleinopathy, this sequestration has been shown to play a role in preventing toxicity and disease progression (Lindstrom et al., 2017; Lin et al., 2019). Astrocytes degrade absorbed α-syn oligomer via the autophagy lysosomal degradation pathway, but remaining intracellular deposition of α-syn causes mitochondrial dysfunction and cell death (Lindstrom et al., 2017). Another study showed that overexpression of wild-type and disease associated mutant (A30P and A53T) α-syn in immortalized astrocytes decreased LC3-II and increased p62 protein levels, suggesting the suppression of autophagy. In addition, mitochondrial membrane potential loss and increased parkin expression were evident in mutant α-syn expressing astrocytes (Erustes et al., 2018). Moreover, a recent study demonstrated that overexpression of mutant α-syn (A30P and A53T) in primary rat astrocytes induced an unfolded protein response through the PERK/eIF2alpha signaling pathway. In particular, dysfunction of the ER-Golgi compartment in astrocytes overexpressing wild type α-syn can inhibit neurite growth by reducing GDNF levels (Liu et al., 2018). In another study, α-syn was transfected into ONL-t40 cells, an oligodendroglial cell line. This study showed that α-syn overexpression induces mitochondrial dysfunction, impaired autophagic flux, and the formation of α-syn aggregates in ONL-t40 cell lines (Pukass et al., 2015). It is clear that mitochondrial impairment is implicated in α-syn-induced glial cell death, but the molecular mechanisms of glial mitochondrial dysfunction and cell death in α-Synucleinopathies are not fully understood.

### Inflammation

Mitochondrial dysfunction is associated with the induction of the inflammatory response. Mitochondrial impairment increases mtROS production, extracellular ATP levels and mtDNA release, all of which induce a vicious inflammatory response cycle that exacerbates mitochondrial dysfunction (Lopez-Armada et al., 2013).

Among the various putative factors contributing to PD etiology, the neuroinflammatory mechanism serves as a major contributor. Increased pro-inflammatory cytokines (including IL-1β, IL-6, TNF, and IFN-γ) have been observed in postmortem brains and cerebrospinal fluid from PD patients (Mogi et al., 1994a, b), moreover, the blood serum of PD patients also showed increased concentration of inflammatory cytokines (including IL-2, IL-6, TNF-α, and IFN-γ; Reale et al., 2009). Neuroinflammation is primarily mediated by the activation of glial cells and is accompanied by the production of inflammatory cytokines. Microglia have traditionally been considered to play a major role in the immune response in the CNS (Soulet and Rivest, 2008). Pathological α-syn aggregation in PD causes microglial activation, which is known to strongly induce microglial neuroinflammatory responses in the brain (Zhang et al., 2005). Microglia that absorb α-syn aggregates via phagocytosis promote NADPH oxidase activation and ROS production, leading to the secretion of pro-inflammatory neurotoxic cytokines (Zhang et al., 2005; Walter and Neumann, 2009) and chemokines (Fellner et al., 2013; Roodveldt et al., 2013). In addition, during the inflammatory response, changes in mitochondrial metabolism contribute to microglial activation.

The levels of inflammatory cytokines, such as IL-6, and the number of activated microglia are increased in the hippocampus in patients with PD and DLB (Imamura et al., 2005). M1-related cytokines, such as TNF-α (Mogi et al., 1994b) and IL-6 (Muller et al., 1998), were increased in the cerebrospinal fluid of PD patients, and the levels of these cytokines correlated with poor prognosis. A number of studies have shown that α-syn induces the pro-inflammatory response in microglia or macrophage to produce the inflammatory cytokines such as IL-1β, IL-6, and TNF-α (Klegeris et al., 2008; Lee et al., 2009). Inflammatory mediators such as iNOS and COX2 were also upregulated in activated microglia by wild-type α-syn treatment. This result suggested that that CD36 scavenger receptor and downstream kinases are involved in α-syn-mediated microglial activation. (Su et al., 2008). Hoenen et al. (2016) also showed that a disease associated α-syn mutant (A35T) more strongly induced reactive microglia than the human wild-type protein in a transgenic mouse model. The researchers also found that the NF-κB/AP-1/Nrf2 pathway was implicated in A35T α-syn-induced microglial activation via MAPK. In addition, Rannikko et al. (2015) reported that overexpression of exogenous wild type α-syn in primary mouse astrocytes can induce inflammatory responses such as induction of NO synthase and COX-2 expression.

Furthermore, in the Fellner (2013; Fellner et al., 2013) study, purified microglia and astrocytes from wild-type (TLR4 +/+) and TLR4-deficient (TLR4-/-) mice were treated with α-syn (full length soluble, fibrillized, and C-terminally truncated), and phagocytic activity, NF-κB nuclear translocation, cytokine release and ROS production were measured. Treatment of TLR4+/+ glial cells with C-terminally truncated α-syn induced upregulation of phagocytic activity, NF-κB translocation, ROS production, and the release of IL-6, CXCL1, and TNF-α (Fellner et al., 2013). The stefanova (2011; Stefanova et al., 2011) study reported that α-syn clearance was reduced in TLR4 deficiency murine. Moreover, in an animal model study of MSA based on oligodendroglial α-syn overexpression, TLR4 deficiency increased motor impairment, and increased loss of nigrostriatal dopaminergic neurons. However, the role of TLR4 in α-syn-dependent activation of microglial and astrocytes has not been clearly elucidated.

To date, many studies have shown that activated astrocytes also play an important role in neuroinflammation in the aged brain and most neurodegenerative diseases. Activated astrocytes release inflammatory cytokines and chemokines that can cause neuronal damage (Komine et al., 2018; Yu et al., 2018). Although the exact pathogenic mechanism of PD is unclear, accumulating evidence suggests that reactive astrogliosis and astrocyte-mediated neuroinflammation play important roles in the pathogenesis of PD (Wang et al., 2015; Liddelow et al., 2017). In another study, inflammation-related factors such as GFAP, COX-2, iNOS, TNF-α, and IL-1β were increased in primary rat astrocytes by A53T α-syn aggregate treatment via the NF-κB and c-Jun N-terminal kinase signaling pathways, and the upregulation of heat shock protein 70 effectively suppressed the α-syn-induced neuroinflammatory response (Yu et al., 2018).

### Calcium Homeostasis

Calcium is essential for cellular signaling, and calcium homeostasis is important for neuronal integrity because it is involved in neuronal plasticity, synaptic transmission, and cell survival. In neurons, calcium channels are the key signaling elements that regulate the release of biological factors, such as neurotransmitters and hormones, through calcium sensing (Leandrou et al., 2019). Among the causes of α-syn pathology, the breakdown of calcium homeostasis promotes aggregation and abnormal secretion of α-syn (Wojda et al., 2008; Hettiarachchi et al., 2009; Yang et al., 2019). Cell culture model studies suggested that a temporary increase in intracellular calcium concentration induced cytoplasmic α-syn aggregates (Nath et al., 2011; Follett et al., 2013).

Nath et al. (2011) showed that the addition of calcium to the recombinant α-syn monomer *in vitro* triggers oligomer formation in a dose-dependent manner. Besides, in 1321N1 glioma cells expressing α-syn-GFP, when treated with thapsigargin or calcium ionophore to induce a temporary increase in intracellular free calcium, α-syn aggregates in the cytoplasm were significantly increased. Another study show that raised intracellular Ca^2+^ mediated by K^+^ depolarization can lead to cytoplasmic α-syn aggregation (Follett et al., 2013).

Dufty et al. (2007) demonstrated that cleavage of α-syn by calpain I, a calcium-dependent protease, can facilitate the aggregation of α-syn. Moreover, Luth et al. (2014) investigated the relationship between mitochondrial Ca^2+^ stress and α-syn using isolated mitochondria and purified recombinant human α-syn. Interestingly, the mitotoxic effect of α-syn was particularly dependent on electron flow through complex I and the mitochondrial uptake of exogenous Ca^2+^. Consequently, the soluble prefibrillar α-syn oligomer induces several mitochondrial phenotypes (changes in membrane potential, disruption of Ca^2+^ homeostasis, mitochondrial complex I dysfunction, and enhancement of cytochrome c release) that have been observed *in vitro* and *in vivo* models of PD. However, the mechanism by which calcium promotes α-syn aggregation is not clear, and the interaction between calcium and α-syn aggregation in glial cells is not been clearly characterized.

## Conclusion

### Links Between Glial Mitochondrial Dysfunction and Neurodegeneration in α-Synucleinopathies

**FIGURE 2:**
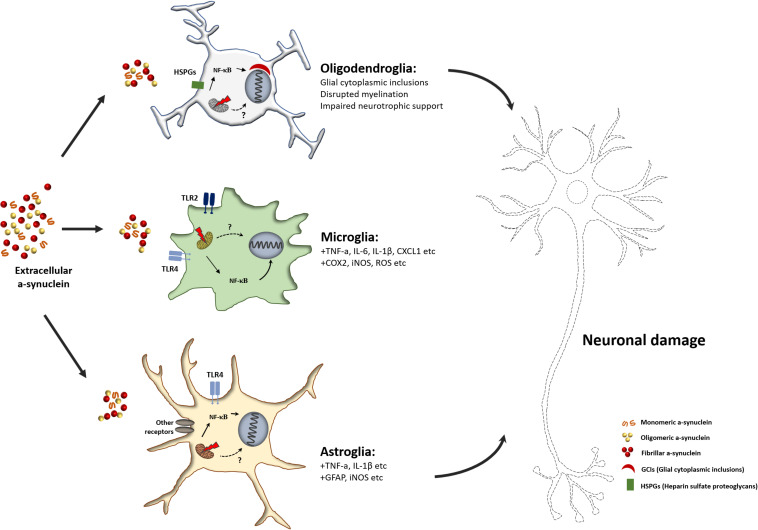
Schematic overview of glia mediated neurodegeneration in α-Synucleinopathies. Unfolded or misfolded α-syn forms α-syn aggregates, and extracellular aggregated α-syn can transfer into glial cells. Internalized α-syn can impair mitochondria in glial cells. Mitochondrial dysfunction caused by α-syn facilitates the pro-inflammatory activation of glial cells. The release of pro-inflammatory factors or neurotoxic cytokines from reactive glial cells causes neuronal damage.

Many *in vitro* and *in vivo* animal studies have focused on neuron-to-neuron propagation of α-syn aggregates (Lee et al., 2012; Dehay et al., 2016). However, studies on α-syn in the context of neuronal dysfunction and studies on the association of α-syn accumulation and propagation in glial cells have been largely overlooked. Some recent studies have provided evidence for the pathogenic response of glial cells to neuron-derived α-syn aggregates. α-syn secreted from neurons induces mitochondrial impairment in microglia, and α-syn-induced mitochondrial dysfunction can activate the microglia-mediated neuroinflammatory response (Figure 2). Pro-inflammatory M1 microglia secrete neurotoxic agents that cause neurodegeneration. In addition, it has been found that the neurotoxic response of microglia is mediated by the activation of TLR2, a receptor for neuron-derived α-syn (Kim et al., 2013, 2016). Similar to microglia, pathogenic α-syn-treated astrocytes activate pro-inflammatory responses, including the production of pro-inflammatory cytokines and chemokines (Lee et al., 2010b). Although mitochondrial dysfunction in glial cells is one of the major contributors to neuroinflammation, which causes neurodegeneration in α-Synucleinopathies, the molecular mechanism underlying α-syn-induced mitochondrial impairment is not fully understood. Thus, further studies are warranted to elucidate the non-cell autonomous neurotoxic crosstalk between glial cells and neurons, and this crosstalk mechanism may be a promising target for α-Synucleinopathy-associated neurodegenerative disease treatment.

## Author Contributions

SL, YK, SK, MJ, and Y-MJ provided ideas for the project and participated in data collection. Y-MJ, YK, and H-JK wrote the manuscript. All authors read and approved the final manuscript.

## Conflict of Interest

The authors declare that the research was conducted in the absence of any commercial or financial relationships that could be construed as a potential conflict of interest.
