# Supplementary material for: The Role of Glial Mitochondria in α-Synuclein Toxicity
Source: Front Cell Dev Biol. 2020 Nov 11;8:548283. doi: 10.3389/fcell.2020.548283 (PMC7686475; doi:10.3389/fcell.2020.548283)
Supplement: Supplementary file 1 [file Table_1.DOCX]

**Table 1. Summary of pathological features induced by α-syn in Glia**

| **Cell type** | | **α-syn isoform** | **Pathological feature** | **References** |
| --- | --- | --- | --- | --- |
| Oligodendroglia | Primary rat oligodendroglia | -Wild type α-syn | -Neuron secreted soluble α-syn oligomer induce oligodendroglial accumulation of α-syn aggregates | Kisos et al. 2012 |
|  | Rat ONL-t40 | -Wild type α-syn | -Impairment of the autophagic flux induced by mitochondrial dysfunction promotes a-syn aggregate formation | [Pukass et al. 2015](#_ENREF_156) |
|  | Rat OLN-93 / in vivo | -Monomeric  -Oligomeric  -Fibrillar α-syn | -Oligodendroglia are prone to the accumulation of neuron-derived α-synuclein | Reyes et al. 2014 |
|  | In vivo | -Wild type α-syn | -Oligodendroglial α-syn overexpression is critical for the increased susceptibility to oxidative stress | Stefanova et al. 2005 |
|  | In vivo  (oligodendroglial α-syn Tg mice) | -Wild type α-syn | -TLR4 ablation enhances neurodegeneration in a transgenic MSA mouse model | Stefanova et al. 2011 |
| Astroglia | Primary rat  / co-culture system | -Monomer  -Oligomer  -Fibrillar α-syn | -Oligomeric α-syn induces mitochondrial dysfunction and increased hydrogen peroxided generation  -Fibrillar α-syn enhanced the secretion of pro-inflammatory cytokines  -All α-syn species induce astroglia activation that can lead to neuronal death | Chavarria et al. 2018 |
|  | Primary rat  (immortalized cells) | -Wild-type  -Mutant (A30P and A53T) α-syn | - α-syn inhibits autophagic degradation pathway  - Loss of mitochondrial membrane potential | Erustes et al. 2018 |
|  | In vivo  (TLR4^+/+^, TLR4^-/-^ Tg mice) | -Full length soluble  -Fibrillar  -C-terminally truncated α-syn | -α-syn treatment increases ROS release, and secretion of TNF- α, IL-6, and CXCL1 | Fellner et al. 2013 |
|  | Primary rat | Wild type α-syn | -Exposure to α-syn increases the secretion of inflammatory cytokines such as IL-1, IL-6, CC, CXC, and CX3C-type chemokines | Lee et al. 2010 |
|  | Primary mice / co-culture system | -Oligomeric α-syn | -Persistent intracellular deposition of α-syn causes mitochondrial dysfunction and cell death | Lindström et al. 2017 |
|  | Primary rat | -Mutant (A30P and A53T) α-syn | -Induction of unfolded protein response through the PERK/eIF2alpha signaling pathway  -Induction of apoptosis and Golgi injury mediated by CHOP  -Astroglia overexpressing α-syn showed inhibited neurite growth by reducing GDNF levels | Liu et al. 2018 |
|  | Human 1321N1 astrocytoma | Wild type α-syn | -Cytoplasmic α-syn aggregates increased significantly by calcium treatment | Nath et al. 2011 |
|  | Human ESC-derived astrocytes | Oligomer | -Astroglia directly contact and actively deliver aggregated α-syn to nearby astroglia through tunneling nanotubes  -Excess α-syn oligomer induce ER swelling and mitochondrial dysfunction | Rostami et al. 2017 |
|  | Human U373 astrocytoma | -Wild type  -C-terminal truncated α-syn | -Overexpression of α-syn induces apoptotic cell death and increased susceptibility to oxidative stress | Stefanova et al. 2001 |
| Microglia | Primary mice | -Wild type α-syn | -Neuronal α-syn activates pro-inflammatory microglia | Choi et al. 2020 |
|  | In vivo  (TLR4^+/+^, TLR4^-/-^ Tg mice) | -Full length soluble  -Fibrillar  -C-terminally truncated α-syn | -Increase microglial phagocytic activity  -C-terminally truncated α-syn as the most potent inductor of TLR4-depenet glial activation | Fellner et al. 2013 |
|  | Primary mice | -Wild type  -Mutant (A53T, A30P and E46K) α -syn | -A35T α-syn induces microglial activation more strongly than other α-syn proteins | Hoenen et al. 2016 |
|  | BV2 | -Wild type  -Mutant (A30P and A53T) α-syn | -Exposure to α-syn increased the expression of Cox-2 protein level, the secretion of pro-inflammatory cytokines (TNFa and IL-6), and nitric oxide production  -Impaired phagocytosis, decreased protein level of LAMP-1 | Rojanathammanee et al. 2011 |
|  | Primary mice | -Wild type α-syn | -α-syn-priming increased the secretion of chemokine MCP-1/CCL2 and IP-10/CXCL10, respectively  -Pam3(TLR2/1)- and ssRNA(TLR7)-further affects microglial activation, causing increased TNF-α secretion and decreased IL-13 levels | Roodveldt et al. 2013 |
|  | BV2 | -Wild type α-syn | -α-syn clearance is mediated by the TLR4-dependent pathway | Stefanova et al. 2011 |
|  | Primary mice | -Wild type α-syn | -α-syn-mediated microglial activation increases the generation of pro-inflammatory molecules (TNF-α, IL1β, IL6, COX2, NOX2 and iNOS). | Su et al. 2008 |
|  | Primary rat / co-culture system | -Wild type α-syn | -Activation of microglial phagocytosis, NADPH oxidase activation, and ROS production  -Microglia enhanced the α-syn-mediated dopaminergic neuronal loss | Zhang et al. 2005 |
